# A 5-year mortality-prediction model for patients with stomach cancer, based on the Korean nationwide health insurance claim database

**DOI:** 10.1097/MD.0000000000049360

**Published:** 2026-06-19

**Authors:** Joungyoun Kim, Yong-June Kim, Da-Hye Son, Yong-Hoon Kim, Jeongsook Kim, Hee-Taik Kang

**Affiliations:** a Department of Artificial Intelligence, University of Seoul, Seoul, Republic of Korea; b Department of Urology, Chungbuk National University Hospital, Cheongju, Republic of Korea; c Department of Urology, College of Medicine, Chungbuk National University, Cheongju, Republic of Korea; d Department of Family Medicine, Gangnam Severance Hospital, Yonsei University College of Medicine, Seoul, Republic of Korea; e Department of Biostatistics and Computing, Yonsei University Graduate School, Seoul, Republic of Korea; f Department of Family Medicine, Severance Hospital, Yonsei University College of Medicine, Seoul, Republic of Korea; g Department of Family Medicine, Institute for Innovation in Digital Healthcare, Yonsei University, Seoul, Republic of Korea.

**Keywords:** area under curve, probability, stomach neoplasms, survival, survival rate

## Abstract

Stomach cancer remains a significant health concern in Korea despite a declining trend in incidence and mortality rates. The aim of this study was to develop a predictive model for the 5-year survival of patients with stomach cancer that is particularly useful in the primary-care setting. This retrospective cohort study included 4156 individuals diagnosed with stomach cancer from 2005 to 2016 and registered in the Korean National Health Insurance Database. The outcome variable was the probability of survival for at least 5 years. Potential confounders affecting mortality were considered, including age, body mass index, systolic blood pressure, lifestyle factors, the Charlson comorbidity index (CCI) score, and laboratory test results. A Cox proportional-hazards regression model was employed to develop the prediction model. The predictive performance was measured using the mean area under the receiver operating characteristic curve after 10-fold cross-validation. The mean 5-year survival probabilities were 0.888 for men and 0.892 for women. For men with stomach cancer, age, body mass index, glucose concentration, smoking status, alcohol consumption, physical activity, total cholesterol concentration, gamma-glutamyl transferase concentration, and the CCI score were significant predictors of mortality. In contrast, for women, only age and the CCI score were significant predictors. The mean areas under the receiver operating characteristic curve after 10-fold cross-validation were 0.699 for men and 0.717 for women. The 5-year survival-probability model had a moderately good predictive performance. It may be used to predict the probability of death for individuals who are diagnosed with stomach cancer.

## 1. Introduction

According to Cancer Statistics in Korea, malignant neoplasms are the number 1 cause of death in Korea even though cancer mortality rates have decreased since 2002.^[1]^ Globally, the disease burden of malignant neoplasms has increased.^[2]^ Although its incidence and mortality has declined in many countries for the past few decades,^[2]^ stomach cancer is the fifth most common cancer and the fourth leading cause of cancer-related mortality globally.^[3]^ The age-standardized stomach cancer incidence per 1,00,000 persons in the Korean population has decreased to 25.7 in 2020.^[1]^ The age-standardized incidence for stomach cancer was the fifth highest among all cancers in Korea. However, it ranked second among men, after lung cancer, in 2020, and it was the most common cancer among men before 2020.^[1,4]^ As with the incidence, the mortality rate from stomach cancer has decreased in Korea. These decreasing trends may be partly attributed to the national cancer-screening program, The Korean National Health Insurance Service (NHIS) runs the program, which is focused on 5 types of cancer (stomach, colorectum, liver, breast, and uterine cervix) among high-risk groups.^[5]^ The stomach cancer-screening program involves the use of endoscopy or a barium enema, which has likely contributed to the improved survival rate for stomach cancer.

The prognosis of patients with stomach cancer differs according to age at diagnosis, pathological stage, and lifestyle after active cancer treatment.^[6,7]^ The 5-year relative survival rate of patients in Korea diagnosed with stomach cancer from 2016 to 2020 was 78.0% overall (78.9% for men and 76.0% for women).^[1]^ The 5-year relative survival rates of patients with localized, regional, and distant metastatic stomach cancer were 97.5%, 62.3%, and 6.7%, respectively.^[7]^ This indicates that the stage of stomach cancer may be an important factor in determining 5-year relative survival. However, various factors, such as age; body mass index (BMI); lifestyle factors, including smoking, alcohol consumption, and physical activity; and comorbidities, are associated with survival in patients with cancer.^[8-10]^ If the survival probability of such patients can be predicted based on their health checkup data and medical records, their doctors would be able to provide them with health care appropriate for their life expectancy.

Thus, we aimed to develop a survival-prediction model for patients with stomach cancer that may support risk stratification, prognostic discussion, and referral planning in the primary care setting, where detailed cancer-specific clinical information is often unavailable. The model we developed may be useful in predicting the 5-year survival probability in the primary care setting without knowledge of cancer stage, histological results, or treatment received.

## 2. Materials and methods

### 2.1. Data source

This retrospective cohort study was performed using the Korean National Health Information Database. Almost all Koreans are subscribed to the Korean NHIS, with the exception of Medicaid beneficiaries who have very low household incomes. The Korean NHIS collects insurance information (type, premium, and residential area); information regarding medical use, including hospital visits, medical records (such as prescriptions, procedures, and diagnostic codes), and medical expenses; death-related information (date and cause of death) from death certificates; and data from health checkups based on the national health-screening program. The Korean NHIS provides adults over 40 years of age with biennial health checkups during which the following are analyzed: anthropometric data; blood pressure; lifestyle behaviors, such as smoking, alcohol, and physical activity; medical history; and laboratory test results, including fasting glucose, alanine aminotransferase (ALT), gamma-glutamyl transferase (GGT), and total cholesterol concentrations. The Korean NHIS stores and releases these data for research purposes only. The last date when we accessed for this research purpose was April 1, 2024.

The Institutional Review Board of Severance Hospital, Yonsei University Health System approved this study (IRB No. 4-2023-0707). This research complied with the tenets of the 1964 Helsinki Declaration. Informed consent was waived because this study used de-identified administrative data provided by the NHIS and involved no direct contact with participants.

### 2.2. Study population and definition of malignant neoplasms

**Figure 1.**
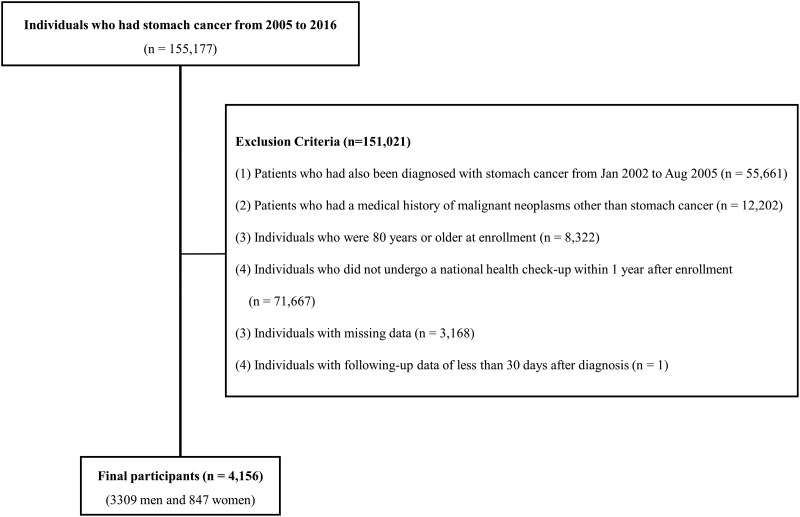
Flowchart of inclusion and exclusion.

Figure 1 presents the flow of participants in this study, according to the inclusion and exclusion criteria. The database contains 1,55,177 individuals who had stomach cancer from 2005 to 2016. Of these, individuals who met any of the following criteria were excluded: patients who had also been diagnosed with stomach cancer from January 2002 to August 2005 (n = 55,661); patients who had a medical history of malignant neoplasms other than stomach cancer (n = 12,202); individuals who were 80 years or older at enrollment (n = 8322); individuals who did not undergo a national health checkup within 1 year before being newly diagnosed with stomach cancer (n = 71,667); individuals with missing data (n = 3168); and individuals with follow-up data of <30 days after diagnosis (n = 1). Ultimately, 4156 individuals (3309 men and 847 women) were included in the final analyses.

Patients with malignant neoplasms were defined as those assigned the special codes V193 or V194 in their medical records. Special codes are normally used for severe refractory diseases, such as cardiovascular diseases or kidney failure, and rare diseases, such as congenital anomalies or severe metabolic diseases, in addition to malignant neoplasms.^[11]^ These special diseases are strictly monitored because the Korean NHIS covers more of the hospital bills of such patients than those of patients with more common or less refractory diseases. Among these special codes, V193 and V194 indicate malignant neoplasms. The types of malignant neoplasms are defined according to the combination of the V193 or V194 codes and the main diagnostic codes, which are based on the International Classification of Diseases (ICD)-tenth edition (ICD-10) codes for cancer (C00-C99). Patients with stomach cancer were defined as those with both the C16 ICD code and the V193 or V194 special code. The index date for follow-up was defined as the date of stomach cancer diagnosis.

### 2.3. Prediction variables

Variables obtained from health checkups and personal medical records were selected as potential predictors of death of patients with stomach cancer. Variables included in the multivariable model were prespecified a priori based on prior literature and clinical relevance, rather than selected through data-driven variable selection procedures. Baseline predictor variables, including laboratory test results and anthropometric measurements, were obtained from a national health checkup performed within 1 year before the index date. When multiple health checkups were available, the examination closest to the diagnosis date was selected. This prediagnostic definition was used to ensure that all predictors preceded follow-up and to minimize the influence of changes that may occur after cancer diagnosis or treatment. The continuous variables were as follows: age, BMI, systolic blood pressure (SBP), and laboratory test results (fasting glucose, ALT, GGT, and total cholesterol concentrations). The categorical variables were as follows: health behaviors (smoking, alcohol consumption, and physical activity), economic status (estimated from the insurance premium), and the Charlson comorbidity index (CCI) score. Regarding smoking status, patients were categorized as never smokers (those who had never smoked), former smokers (those who had smoked in the past but not at the time of the checkup), and current smokers (those who smoked at the time of the checkup). Regarding alcohol consumption, patients were classified into rare, moderate, and heavy drinkers. Rare drinkers were individuals who consumed alcohol 3 times or less per month; moderate drinkers were those who consumed alcohol once to 4 times per week; and heavy drinkers were those who consumed alcohol 5 or more times per week. Physical activity was divided into low, moderate, and high levels. A low level was defined as engaging in physical activity less than twice per week; moderate, as 3 or 4 times per week; and high, as 5 times or more per week. In terms of household income, patients were categorized into 5 groups according to insurance premium.

The CCI is used to categorize patients according to their comorbidities based on the ICD codes recorded in administrative data.^[12]^ Each comorbidity has weighted values from 1 to 6 based on the adjusted risk of death or resource use associated with it. The sum of all the weights provides a single comorbidity score for each patient. A score of 0 means that no serious comorbidities were detected. A higher CCI score indicates a greater likelihood that a condition will result in death or higher medical resource use.^[13]^ In this study, patients were categorized into 4 groups based on the CCI score (0, 1, 2, and 3 or more).

### 2.4. Statistical analysis

Continuous variables (age, BMI, SBP, fasting glucose concentration, total cholesterol concentration, ALT concentration, and GGT concentration) were presented as the mean ± standard deviation. Categorical variables (cigarette smoking, alcohol consumption, physical activity, household income, and CCI score) were expressed as the number of patients (percentage).

A Cox proportional-hazards regression model was used to develop a prediction model for the 5-year mortality of patients with stomach cancer.^[14,15]^ Patients were censored at the date of their last clinic visit if they did not die during the study period. Potential risk factors considered in this analysis were age, BMI, SBP, laboratory results (fasting glucose, total cholesterol, ALT, and GGT concentration), smoking status, alcohol consumption, physical activity, economic status, and CCI score. These variables, except for the CCI score, were collected in the national health checkup program.

The 5-year mortality probability for a patient with stomach cancer can be estimated using the following equation:


$$P\left(survival\;\;\;time\leq t\right)=1-S_{0}\left(t\right)exp^{\beta_{1}\left(x_{1}-M_{1}\right)+\beta_{2}\left(x_{2}-M_{2}\right)+\cdots +\beta_{p}\left(x_{p}-M_{p}\right)}.$$

*S*_0_(*t*) is the survival rate at the mean value of the risk factor at time *t*. In this study, the 5-year survival rate *S*_0_(5) was estimated as 0.888 for men and 0.892 for women. Moreover, β_*j*_ and *M*_*j*_ (*j* = 1, 2, …,*p* = 12) are the regression coefficients in the Cox proportional-hazards model and the sample mean of the *j*th risk factor, respectively.

We evaluated the performance of the model by using 10-fold cross-validation. *K*-fold cross-validation is used to divide the data into *K* folds of the same size, fit the prediction model with *K* − 1 folds as the training set, and measure the predictive performance of the fitted model with the remaining fold as the test set. Every fold is used as a test set once, and in this study, the predictive performance was measured using the average area under the receiver operating characteristic curve (AUC).^[16]^

## 3. Results

Table 1 presents the patients’ characteristics at baseline. The number of person-years of follow-up was 22,700 for men and 18,102 for women, with a median follow-up duration of 13.84 years. The mean age of men and women was 56.20 and 56.96 years, respectively. The mean BMI was 23.14 kg/m^2^ for men and 22.51 kg/m^2^ for women. The percentages of current smokers and heavy drinkers were 22.5% and 11.0%, respectively, among men and 1.5% and 1.1%, respectively, among women. The proportions of individuals who were engaged in moderate and high physical activity were 15.3% and 12.0%, respectively, among men and 11.1% and 12.8%, respectively, among women. Patients with multiple comorbid diseases (CCI ≥ 3) comprised 8.8% and 6.3% of men and women, respectively.

**Table 1:** Participants’ characteristics at baseline.

	Male	Female	*P*-value
Number	3309	847	
Age, yrs	56.20 ± 10.58	56.96 ± 12.31	.071
Body mass index, kg/m^2^	23.14 ± 2.89	22.51 ± 3.31	<.001
Systolic blood pressure, mm Hg	123.53 ± 15.31	120.37 ± 16.34	<.001
Fasting glucose, mg/dL	100.81 ± 27.07	95.71 ± 25.36	<.001
Alanine aminotransferase, IU/L	27.00 ± 26.52	21.97 ± 16.21	<.001
Gamma-glutamyl transferase, IU/L	41.63 ± 58.25	22.12 ± 23.60	<.001
Total cholesterol, mg/dL	184.52 ± 35.69	191.44 ± 35.97	<.001
Smoking status, n (%)			<.001
Never smokers	1654 (50.0%)	821 (96.9%)	
Former smokers	911 (27.5%)	13 (1.5%)	
Current smokers	744 (22.5%)	13 (1.5%)	
Alcohol consumption, n (%)			<.001
Rare	1881 (56.8%)	771 (91.0%)	
Moderate	1065 (32.2%)	67 (7.9%)	
Heavy	363 (11.0%)	9 (1.1%)	
Physical activity, n (%)			.008
Low	2405 (72.7%)	645 (76.2%)	
Moderate	506 (15.3%)	94 (11.1%)	
High	398 (12.0%)	108 (12.8%)	
Household income, n (%)			<.001
0th to 20th percentile	626 (18.9%)	223 (26.3%)	
21st to 40th percentile	567 (17.1%)	178 (21.0%)	
41st to 60th percentile	681 (20.6%)	163 (19.2%)	
61st to 80th percentile	792 (23.9%)	169 (20.0%)	
81st to 100th percentile	643 (19.4%)	114 (13.5%)	
Charlson comorbidity index, n (%)			<.001
0	1333 (40.3%)	390 (46.0%)	
1	1232 (37.2%)	300 (35.4%)	
2	454 (13.7%)	104 (12.3%)	
≥3	290 (8.8%)	53 (6.3%)	

Values are means ± standard deviations, unless otherwise indicated.

Table 2 summarizes the survival rates of patients with stomach cancer after the initial diagnosis. The mean survival probability at 1, 3, and 5 years after the stomach cancer diagnosis was 0.983, 0.927, and 0.888, respectively, for men and 0.988, 0.927, and 0.892, respectively, for women. As the time since cancer diagnosis increased, the survival rate decreased. The β-coefficients and hazard ratios in the stomach cancer survival models are presented in Table 3. Age and a CCI score ≥ 3 were positively associated with mortality among both sexes. BMI, fasting glucose concentration, GGT concentration, and total cholesterol concentration were significantly related to mortality among men. Male current smokers were at a higher risk for mortality, whereas male moderate drinkers and moderate exercisers were at a lower risk, compared to the reference group. Prediction models for the 5-year survival probability were constructed from the mean values in Table 1, the 5-year survival rate in Table 2, and the β-coefficients in Table 3. The final prediction model included age, comorbidity burden, lifestyle factors, and selected laboratory measurements as key predictors of 5-year mortality. Detailed model formulas and an Excel-based 5-year mortality prediction calculator are provided in Supplementary Appendix 1 and 2, Supplemental Digital Content 1 to ensure transparency and reproducibility, and an illustrative example of the spreadsheet calculator is shown in Figure S1, Supplemental Digital Content 2.

**Table 2:** Survival rate among patients with stomach cancer.

Sex	Men			Women		
Time since diagnosis	1 yr	3 yr	5 yr	1 yr	3 yr	5 yr
Number at risk	3252	3065	2936	835	781	752
Number of deaths	56	186	128	10	52	29
Survival rate (95% confidence interval)	0.983 (0.979–0.987)	0.927 (0.918–0.936)	0.888 (0.877–0.899)	0.988 (0.981–0.995)	0.927 (0.909–0.944)	0.892 (0.872–0.913)

Number of deaths indicates deaths within each interval.

**Table 3:** Hazard ratios and 95% confidence intervals for death among patients with stomach cancer.

	Men		Women	
	Coefficient	HR (95% CI)	Coefficient	HR (95% CI)
Age, yr	0.0756	1.079 (1.070–1.087)	0.0692	1.072 (1.054–1.089)
Body mass index, kg/m^2^	−0.0656	0.937 (0.914–0.959)	−0.0344	0.966 (0.923–1.011)
Systolic blood pressure, mm Hg	0.0021	1.002 (0.998–1.006)	−0.0061	0.994 (0.985–1.003)
Fasting glucose, mg/dL	0.0026	1.003 (1.001–1.005)	0.0021	1.002 (0.998–1.006)
Alanine aminotransferase, IU/L	−0.0016	0.998 (0.996–1.001)	−0.0041	0.996 (0.985–1.007)
Gamma-glutamyl transferase, IU/L	0.0018	1.002 (1.001–1.003)	0.0023	1.002 (0.998–1.007)
Total cholesterol, mg/dL	−0.0030	0.997 (0.995–0.999)	0.0006	1.001 (0.997–1.005)
Smoking status, n (%)				
Never smokers	0.000	Reference	0.000	Reference
Former smokers	−0.1133	0.893 (0.747–1.068)	0.5267	1.693 (0.584–4.907)
Current smokers	0.3255	1.385 (1.180–1.626)	0.2984	1.348 (0.515–3.528)
Alcohol consumption, n (%)				
Rare	0.000	Reference	0.000	Reference
Moderate	−0.2012	0.818 (0.732–0.968)	0.3205	1.378 (0.748–2.537)
Heavy	−0.0304	0.970 (0.691–1.204)	−0.0373	0.963 (0.131–7.084)
Physical activity, n (%)				
Low	0.000	Reference	0.000	Reference
Moderate	−0.2666	0.766 (0.746–0.940)	−0.5637	0.569 (0.315–1.027)
Heavy	−0.1139	0.892 (0.624–1.088)	−0.2152	0.806 (0.516–1.261)
Household income, n (%)				
0th to 20th percentile (very low)	0.000	Reference	0.000	Reference
21st to 40th percentile (low)	0.1790	1.196 (0.980–1.460)	−0.0223	0.978 (0.608–1.573)
41st to 60th percentile (mid-low)	0.1858	1.204 (0.993–1.460)	−0.0713	0.931 (0.569–1.523)
61st to 80th percentile (mid-high)	0.0289	1.029 (0.846–1.252)	0.4100	1.507 (0.984–2.308)
81st to 100th percentile (high)	−0.1502	0.861 (0.686–1.079)	0.2284	1.257 (0.784–2.014)
CCI, n (%)				
0 (CCI0)	0.000	Reference	0.000	Reference
1 (CCI1)	0.1079	1.114 (0.952–1.304)	0.3441	1.411 (0.998–1.993)
2 (CCI2)	0.1843	1.202 (0.985–1.469)	0.4123	1.510 (0.972–2.348)
≥3 (CCI3)	0.5279	1.695 (1.377–2.087)	0.7245	2.064 (1.225–3.478)

CCI = Charlson Comorbidity Index, CI = confidence interval, HR = hazard ratio.

**Figure 2.**
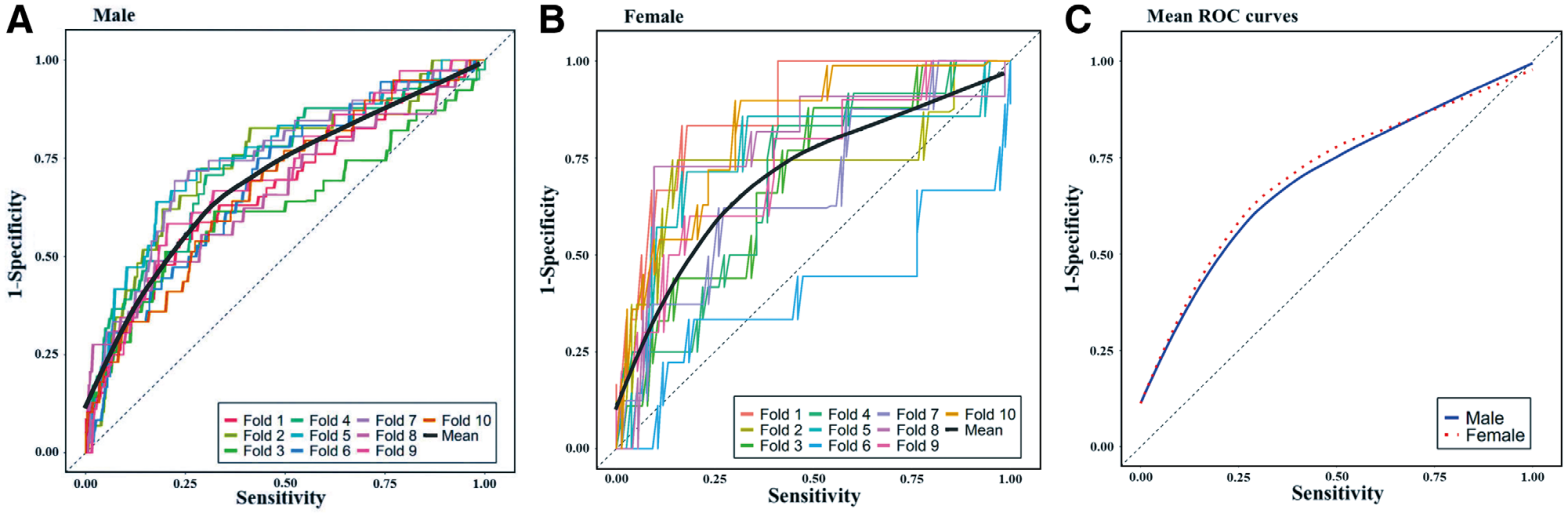
Area under the receiver operating characteristic curves via 10-fold cross-validation (A) male, (B) female, and (C) mean ROC curves. The mean of AUCs: 0.699 for male and 0.717 for female. AUC = area under the receiver operating characteristic curve, ROC = receiver operating characteristic.

The performance of the prediction model was measured via 10-fold cross-validation. Figure 2 displays the 10 receiver operating characteristic curves and the mean curve for men and women separately. The means of AUCs were 0.699 and 0.717 for men and women, respectively. The AUC result of approximately 0.7 suggests that the model has moderately good predictive performance.

## 4. Discussion

In this study, a 5-year survival-probability model for patients newly diagnosed with stomach cancer was developed using a nationally representative cohort.

Koreans are at higher risk of stomach cancer than Westerners and individuals from other East Asian countries.^[17]^ Although the age-standardized stomach cancer incidence rate in Korea has been decreasing over recent decades (from 43.6 per 1,00,000 in 1999 to 25.7 per 1,00,000 in 2020), it remains high.^[1]^ Predicting the survival probability of this common cancer is important for treatment planning. The 5-year relative survival rate of patients with stomach cancer in Korea has also gradually improved,^[1]^ reaching 78.0% for those diagnosed from 2016 to 2020. However, the mean 5-year survival rate was 88.8% for men and 89.2% for women in this study, higher than that reported by Statistics Korea. A reason for the possible overestimation in this study is that the sample comprised individuals who participated in the national health checkup within 1 year before their cancer diagnosis. Therefore, patients in the study sample were all sufficiently healthy to participate in the national health checkup, causing a selection bias.

Woo et al constructed a 5-year survival model for the prognosis of patients who underwent a gastrectomy for stomach cancer, as registered in Asian databases.^[18]^ Their model has the advantage of including pathological data and data on lymph node involvement and surgical type. Prediction models have also been developed for patients who have undergone surgical resection for stomach cancer.^[19,20]^ However, our prediction model was developed based on data obtained from national health checkups. For this reason, even nonmedical personnel and primary-care doctors, who do not have access to detailed information such as pathology results, TNM stage, or type of treatment (type of surgery, chemotherapy, and radiation therapy), can easily use this model. Previous prediction models based on surgical or pathological data have reported AUC values ranging from approximately 0.75 to 0.85, such as the model by Woo et al and more recent approaches by Wang et al. In contrast, our model achieved AUC values of 0.699 in men and 0.717 in women using only routinely available health checkup data, highlighting the trade-off between predictive performance and feasibility in less specialized clinical settings. However, given the moderate discriminative performance of the model, it should not be interpreted as a tool for definitive individual-level clinical decision-making. Rather, it is intended to serve as a pragmatic aid for risk stratification and initial prognostic discussion in primary care settings. In women, the limited number of significant predictors likely reflects both the smaller sample size and the low prevalence of lifestyle risk factors, such as smoking and heavy alcohol consumption, rather than a lack of clinical relevance of these factors.

This study has several strengths compared with previous studies. It was designed based on nationwide insurance-claim data. We used various confounding factors that may influence survival but are useful in the primary-care setting. Social determinants of health, such as economic status, should be considered when a patients’ survival probability is determined. Economic status is one of the major determinants of medical service use and accessibility, which is why it was considered in our formula. Individuals with a low economic status have a higher cancer incidence and mortality than wealthy persons.^[21-23]^ The CCI score was previously used in a risk-prediction formula, as it was in ours, as it is a measure of comorbid conditions related to patient death or medical resource utilization.^[13]^ In addition, extensive anthropometric data and laboratory results were included in the current formulae. Thus, the risk-prediction models that we developed offset the effects of unexpected confounders on survival probability.

However, several limitations should be considered when the results of this study are interpreted. First, stage, histological type, and treatment type were not available in the National Health Insurance database. Second, the survival rate relative to the time of cancer diagnosis was not reflected because of the long study period (2005–2016) and the improvement in the 5-year relative survival rate of patients with stomach cancer (67.4% in 2006–2010 and 78.0% in 2016–2020).^[1]^ Third, there may be selection bias. Because one of the inclusion criteria was completion of a national health checkup within 1 year before cancer diagnosis, the study population may represent a relatively healthier subgroup of patients. This selection characteristic may have contributed to the higher observed 5-year survival probabilities compared with national statistics and limits the generalizability of the model to the broader and potentially frailer stomach cancer population. At the same time, it should be noted that in Korea, the Ministry of Health and Welfare almost freely provides a nationwide health screening program every 2 years for adults, resulting in broad population-level access to routine health checkups. Because access to health checkups is broad and participation is actively encouraged at the population level, health checkup attendance is not limited only to individuals who are highly health-conscious. Therefore, while some degree of selection bias is unavoidable, the extent of selection related to health checkup participation may be less pronounced than in countries without a universal screening system. Fourth, no external validation cohort was analyzed because the number of patients with stomach cancer was too small. In addition, model evaluation was limited primarily to discrimination, and formal calibration assessment, verification of proportional hazards assumptions, and assessment of potential nonlinearity of continuous predictors were not performed. However, to mitigate the lack of a validation cohort, we used 10-fold cross-validation to fit the model. Given their impact on interpretation, these limitations warrant particular attention. The model’s applicability is restricted to patients with available health checkup data, and the absence of calibration assessment and external validation limits its generalizability and precludes definitive clinical decision-making. Future studies should perform formal calibration and validate the model in independent external cohorts before clinical implementation.

In conclusion, we developed a 5-year mortality prediction model for patients with stomach cancer using nationwide health checkup and claims data. Given its moderate predictive performance, this model should be interpreted as a supportive risk stratification tool rather than a definitive instrument for individual-level clinical decision-making. This model, based on routinely available health checkup data, may serve as an initial risk-stratification tool in primary care to facilitate prognostic discussions and referral planning. Its moderate performance and selection bias highlight the need for further validation in broader cohorts before clinical implementation.

## Author contributions

**Conceptualization:** Hee-Taik Kang.

**Data curation:** Yong-Hoon Kim, Jeongsook Kim.

**Formal analysis:** Yong-Hoon Kim, Jeongsook Kim.

**Funding acquisition:** Yong-June Kim.

**Investigation:** Yong-Hoon Kim, Jeongsook Kim, Yong-June Kim, Hee-Taik Kang.

**Methodology:** Joungyoun Kim, Da-Hye Son, Yong-June Kim, Hee-Taik Kang.

**Resources:** Yong-June Kim.

**Supervision:** Joungyoun Kim, Hee-Taik Kang.

**Writing – review & editing:** Da-Hye Son.

**Writing – original draft:** Hee-Taik Kang.






