# Supplementary material for: A 5-year mortality-prediction model for patients with stomach cancer, based on the Korean nationwide health insurance claim database
Source: Medicine (Baltimore). 2026 Jun 19;105(25):e49360. doi: 10.1097/MD.0000000000049360 (PMC13286386; doi:10.1097/MD.0000000000049360)
Supplement: Supplementary file 1 [file medi-105-e49360-s001.docx]

## Supplementary Appendix S1.

## The risk-prediction formulas for 5-year survival in patients with stomach cancer

## For men

(1) $Survival model function for men (SMFM) = 0.0765\times(age - 56.20) - 0.0656\times(BMI - 23.14) + 0.0021\times(SBP - 123.53) + 0.0026\times(fasting glucose concentration - 100.81) - 0.0016\times(ALT concentration - 27.00) + 0.0018\times(GGT concentration - 41.63) - 0.0030\times(total cholesterol concentration - 184.52) - 0.1133\times(former smoker - 0.275) + 0.3255\times(current smoker - 0.225) - 0.2012\times(moderate drinker - 0.322) - 0.0304\times(heavy drinker - 0.110) - 0.2666\times(moderate exerciser - 0.153) - 0.1139\times(heavy exerciser - 0.120) + 0.1790\times(low income - 0.171) + 0.1858\times(mid-low - 0.206) + 0.0289\times(mid-high income - 0.239) - 0.1502\times(high income - 0.194) + 0.1079\times(CCI1 - 0.372) + 0.1843\times(CCI2 - 0.137) + 0.5279\times(CCI3 - 0.088)$

(2) SMFM1 = exp (SMFM)

(3) 5-year survival probability = 1 – 0.888^SMFM1^

## For women

(1) $Survival model function for women (SMFW) = 0.0692\times(age - 56.96) - 0.0344\times(BMI - 22.51) - 0.0061\times(SBP - 120.37) + 0.0021\times(fasting glucose concentration - 95.71) - 0.0041\times(ALT concentration - 21.97) + 0.0023\times(GGT concentration - 22.12) + 0.0006\times(total cholesterol concentration - 191.44) + 0.5267\times(former smoker - 0.015) + 0.2984\times(current smoker - 0.015) + 0.3205\times(moderate drinker - 0.079) - 0.0373\times(heavy drinker - 0.011) - 0.5637\times(moderate exerciser - 0.111) - 0.2152\times(heavy exerciser - 0.128) - 0.0223\times(low income - 0.210) - 0.0713\times(mid-low income - 0.192) + 0.4100\times(mid-high income - 0.200) + 0.2284\times(high income - 0.135) + 0.3441\times(CCI1 - 0.354) + 0.4123\times(CCI2 - 0.123) + 0.7245\times(CCI3- 0.063)$

(2) SMFW1 = exp (SMFW)

(3) 5-year survival probability = 1 – 0.892^SMFW1^
