# Supplementary figures and images for: A 5-year mortality-prediction model for patients with stomach cancer, based on the Korean nationwide health insurance claim database
Source: Medicine (Baltimore). 2026 Jun 19;105(25):e49360. doi: 10.1097/MD.0000000000049360 (PMC13286386; doi:10.1097/MD.0000000000049360)

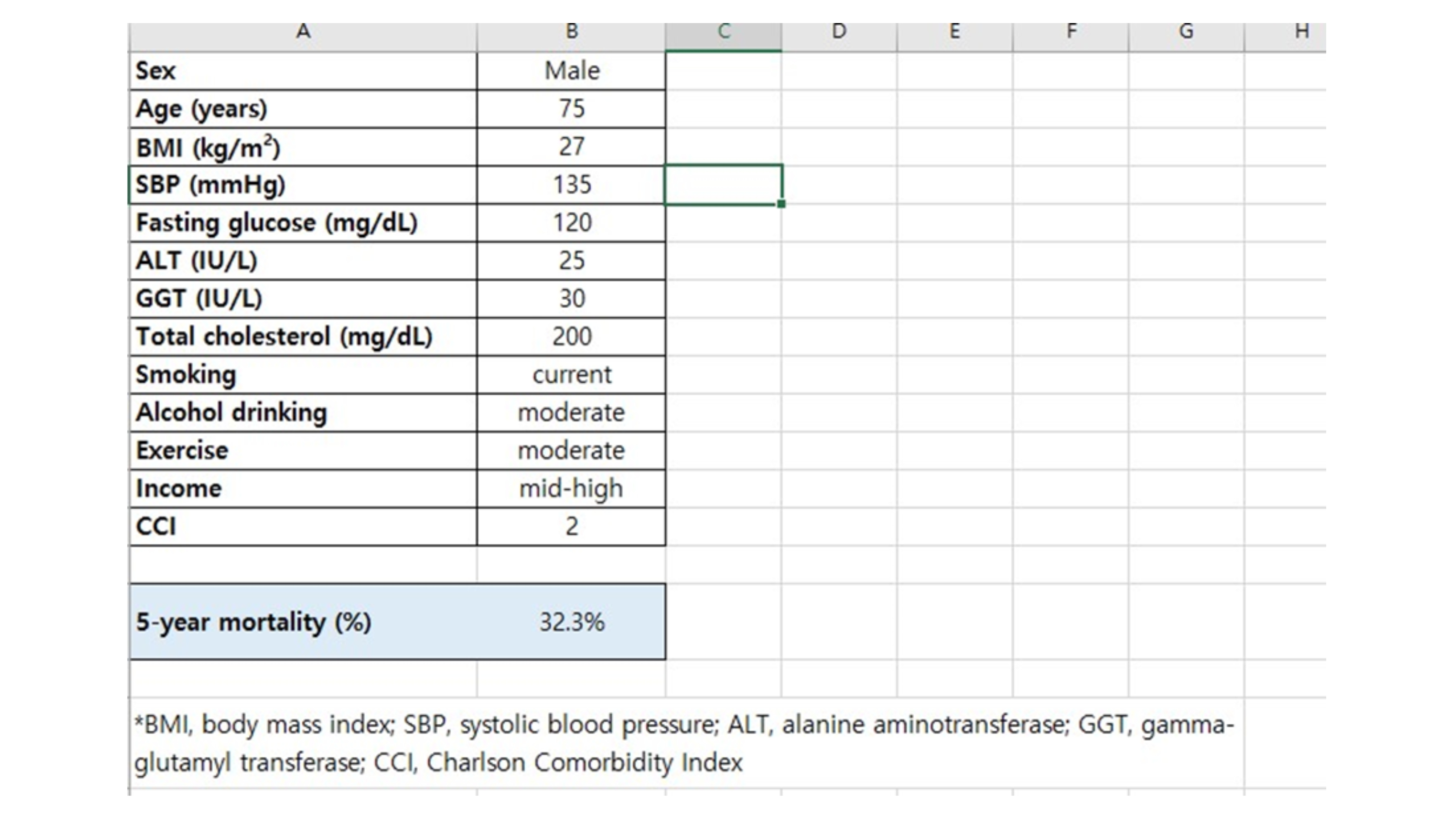

Supplement: Supplementary file 2 [file medi-105-e49360-s002.jpg]
